# Whole-genome sequence of *Bacillus anthracis* linked to the 2023 anthrax outbreak in Zambia

**DOI:** 10.1128/mra.00051-26

**Published:** 2026-06-18

**Authors:** Harvey K. Kamboyi, Misheck Shawa, Flavien N. Bumbangi, Mox M. Kalumbi, Mungunsar Chuluun, Jeremiah B. Chijikwa, Joseph Y. Chizimu, Liyali Libonda, Tuvshinzaya Zorigt, Ladslav Moonga, Joseph Ndebe, Raymond Hamoonga, Tomoe Kitao, Naganori Nao, Masahiro Kajihara, Musso Munyeme, Hirofumi Sawa, Roma Chilengi, Bernard M. Hang'ombe, Hideaki Higashi

**Affiliations:** 1 Directorate of Research, Eden University 679883, Lusaka, Zambia; 2 Department of Medicine and Clinical Sciences, School of Medicine, Eden University 679883, Lusaka, Zambia; 3 Division of Infection and Immunity, International Institute for Zoonosis Control, Hokkaido University 12810, Sapporo, Japan; 4 Hokudai Center for Zoonosis Control in Zambia, Hokkaido University 12810, Lusaka, Zambia; 5 Division of International Research Promotion, International Institute for Zoonosis Control, Hokkaido University 12810, Sapporo, Japan; 6 Institute of Basic and Biomedical Sciences, Levy Mwanawasa Medical University 580010, Lusaka, Zambia; 7 Laboratory of Theriogenology, Graduate School of Veterinary Medicine, Hokkaido University 12810, Sapporo, Japan; 8 Zambia National Public Health Institute, Ministry of Health 576315, Lusaka, Zambia; 9 Microbiology Unit, Paraclinical Studies, School of Veterinary Medicine, University of Zambia 108234, Lusaka, Zambia; 10 One Health Research Center, Hokkaido University 12810, Sapporo, Japan; 11 Public Health Unit, Disease Control Studies, School of Veterinary Medicine, University of Zambia 108234, Lusaka, Zambia; 12 Africa Centre of Excellence for Infectious Diseases of Humans and Animals, School of Veterinary Medicine, University of Zambia 108234, Lusaka, Zambia; 13 Country Multiplier, Capacitating One Health in Eastern and Southern Africa, Lusaka, Zambia; 14 Institute for Vaccine Research and Development, Hokkaido University 12810, Sapporo, Hokkaido, Japan; 15 Division of Molecular Pathobiology, International Institute for Zoonosis Control, Hokkaido University 12810, Sapporo, Japan; Fluxus Inc., Sunnyvale, California, USA

**Keywords:** *Bacillus anthracis*, whole genome sequence, anthrax outbreak, zoonotic disease, Zambia

## Abstract

The 2023 anthrax outbreak in Zambia, spanning from May to November, resulted in 390 livestock and 39 wildlife fatalities. In addition, 834 suspected human cases and 4 fatalities were recorded. This paper reports the genome sequences of the *Bacillus anthracis* isolated from humans and soil during the outbreak.

## ANNOUNCEMENT

*Bacillus anthracis* is a multi-host zoonotic pathogen that causes anthrax and occurs worldwide, especially in resource-limited countries (1–3). In Zambia, anthrax is endemic in Western Province, with annual outbreaks in humans and livestock, and approximately every 5 years in wildlife in the Lower Zambezi and Luangwa Valley (4, 5). The 2023 anthrax outbreak was first detected in humans on 6 May 2023 at the Dengeza Health Post in Sinazongwe district of Southern Province. The outbreak resulted in 834 suspected human cases, including 4 deaths, across 44 of 116 districts in 9 provinces by November 20. Sinazongwe District, a non-endemic region, emerged as the epicenter, accounting for 35% of all cases and two human mortalities. Mortalities in animals included 380 cattle, 6 donkeys, 2 goats, 2 dogs, 31 hippos, 6 buffaloes, 1 elephant, and 1 zebra across 11 districts in the Southern, Western, and Eastern provinces. Here, we report the genome sequences of *B. anthracis* strains linked to the 2023 anthrax outbreak in Zambia.

The strains were isolated from three patients presenting with anthrax carbuncles at Kazungula District Hospital and Livingstone University Teaching Hospital, and one soil sample from a site where a cow died in Livingstone. The suspected samples (wound swabs and soil) were triple-packaged at the collection site and transported to the University of Zambia, School of Veterinary Medicine, BSL3 laboratory for confirmation. The samples were inoculated onto blood agar and incubated at 37°C for 18 h, followed by sub-culturing to obtain pure colonies (6). Presumptive pure *B. anthracis* colonies were inoculated on Brain Heart Infusion agar (Difco, USA) at 37°C for 18 h for genomic DNA extraction using DNAzol Genomic DNA Isolation Reagent (Molecular Research Center, Inc., USA), following the manufacturer’s protocol. For confirmation, multiplex PCR was performed using TaKaRa Ex Taq HS (TaKaRa, Japan) targeting plasmid-encoded *pagA* and *capA* on pXO1 and pXO2, respectively, and *BA1698*, a *B. anthracis*-specific gene encoding a glycosyltransferase on the chromosome. The primer details and thermocycling conditions were as described under MPCR-1 by Zorigt et al. (7). DNA libraries were prepared using NexteraXT (Illumina) and sequenced using NovaSeq X Plus (Illumina) to obtain 150 bp paired-end reads. The reads were first filtered with Trim Galore v0.6.10 (8) using the—paired and—nextera options for adapter trimming and quality filtering. *De novo* assembly utilized Unicycler v0.5.1 (9) using SPAdes.py v3.15.5 (10), followed by quality checks with BBMap v38.18 (11) and QUAST v5.3.0 (12). Further, local BLASTn (13) with the *B. anthracis* Ames ancestor *BA1698*, *pagA* and *capA* (GCA_000008445.1) as query sequences confirmed the presence of these genes in the assembled genomes, matching the targets of multiplex PCR. The genome was structurally and functionally annotated using the NCBI Prokaryotic Genome Annotation Pipeline v6.10 (14). All bioinformatic analyses used default parameters unless otherwise specified. The relevant sequence reads and assembly statistics are shown in Table 1.

**TABLE 1:** Accession numbers and genome characteristics of *Bacillus anthracis* strains linked to the 2023 anthrax outbreak

Strain		KzH23a	KzH23b	LSEv23	LSH23
Source		Human	Human	Soil	Human
Location		Kazungula	Kazungula	Livingstone	Livingstone
NovaSeq reads					
	SRA accession no.	SRR34080554	SRR34080553	SRR34080551	SRR34080552
	No. of read pairs	11,846,323	11,740,786	10,162,242	10,377,575
Assembled genome	GenBank accession no.	GCA_054471315.2	GCA_054471255.2	GCA_054471275.2	GCA_054471295.2
	Genome size (bp)	5,450,737	5,450,739	5,450,874	5,450,876
	No. of contigs	36	36	36	36
	Genome coverage	595×	590×	513×	523×
	N50 (bp)	1,197,642	1,197,642	1,197,642	1,197,642
	L50	2	2	2	2
	G + C content (%)	35.09	35.38	35.24	35.09
	Gene total	5,743	5,745	5,744	5,743
	Gene coding	5,258	5,259	5,259	5,259

## Data Availability

This whole-genome shotgun project has been deposited at DDBJ/ENA/GenBank under BioProject PRJNA1280164 for raw reads and PRJNA1311523 for assembled genomes. Accession numbers for each strain are shown in Table 1. The versions of KzH23a, KzH23b, LSEv23, and LSH23 described in this paper are JBRYHD020000000, JBRYHE020000000, JBRYHF020000000, and JBRYHG020000000, respectively.
